# The Story of a Great Change

**Published:** 1921-03-19

**Authors:** 


### A MENTAL HOSPITAL REVOLUTION.

### The Story of a Great Change.

The story of a great movement is best understood in a single example, and this is particularly true of administrative work. Consequently, if we can turn to a well-written history of the conversion of one asylum to a war hospital, and study, in this single instance, the administrative change, we learn far more than is possible from a mass of general information, an official report of a department, or a summary history of hospitals in the war. There is therefore abundant reason to be grateful for Lieut.-Colonel J. R. Lord's "Story of the Horton (County of London) War Hospital, Epsom,"*

It was on February 9, 1915, that the Asylums Committee decided to offer Horton Mental Hospital to the Army Council, when the Asylum Sub-Committee became the War Hospital Sub-Committee, and the Medical Superintendent the C.O. The evacuation of the mental patients began on March 12, and was completed by April 8, by which time 2,143 patients had been successfully transferred to other institutions. The work involved was very great. Their relations had to be considered, as well as their clothes and capacity to travel, and districts were chosen for them as near as possible to the homes of their families. It was found that "Mr. Hine's pavilion crescentic asylum buildings, with its attached villas," "adapt themselves readily" to the work of a general hospital. Theatres and out-patient accommodation were the chief and unavoidable lacks. In all, we are told, the Army Council took over buildings which cost more than half a million, and over 241 acres of land. One villa was allotted to additional resident medical staff Three villas for chronic cases became nurses' homes, and another a home for night probationers. The two acute hospitals were set apart for special cases. A central post office and a card-index system were installed, and the sheds in the builders' yards became garages. Colonel Lord did not attempt, he said, a complete military administration, though his visit to Queen Alexandra Hospital, Millbank, gave him many useful hints. A well-known change, worthy, however, to be recorded, was the removal of all shutter locks, which were used for the rooms of the female staff and the entrance to the nurses' homes. Spring locks were not removed, but wedged, and the asylum system of locks abolished everywhere except in the ward storerooms and kindred places. Telephones and hydrants were uncovered, and practically all traces of an asylum were removed. The padded rooms were filled with unused asylum stores. Theatres and pack stores had to be added. The adaptations and additions, which cost £5,000, were quickly carried out by the asylum engineer. The Board of Control expected that one-third more beds for hospital cases could be provided than when each institution was filled with mental cases. On this surmise Horton was credited with 2,277 beds; each was provided with 6 ft. of wall or a floor space of 85 sq. ft., as the shape of each room determined. The grand total of beds, including those not in dormitories, eventually was 2,412.

There was, in the administration, "to be just sufficient military element to suit the character of the patients, and no more." Colonel Lord had to perform all the duties of a regimental officer, "except the military training of the soldiers" under his command. He was, therefore, to act in a civilian and in a military capacity. In consequence his table groaned with military manuals (supplied by the War Office), and his new branch of study he found, he says, a good education in itself. He acted on the principle that male assistance as far as possible should be dispensed with. The permanent attendants, who were Army Reservists, had gone, and he foresaw that all able-bodied men would eventually be wanted. This decided him to begin as far as possible with a female staff. Only indispensable male administrators were retained. The unfit civilian orderlies became stretcher-bearers, and helped much when the convoys were arriving. The local practitioners were enrolled, and the already existing Epsom and Ewell War Hospital, in the Grand Stand, became eventually an auxiliary to Horton. The chief resident surgeons and physician were entirely responsible to the C.O. for both surgical and medical treatment, and only the operative work which they could not undertake was allotted to a rota of visiting operating surgeons, who were notified when to attend. Visiting specialists attended on certain days.

The response to advertisements for nurses was so great that an excellent staff was secured. The male attendants who remained became non-resident. Each ward as far as possible became an autonomous administrative unit. It is noteworthy that, though the house steward's department was much enlarged, little or no administrative change was needed. The Clerk became Accountant and Paymaster, with the enormous correspondence in his hands. Lady St. Helier became the Lady Almoner, and she received, acknowledged, and distributed all the gifts, besides choosing those women visitors generally known as "fairy godmothers." Hon. Lieut. and Quartermaster F. S. Marsland, D.C.M., R.A.M.C., was the first officer of the War Hospital to be appointed, and his experience in the South African War was invaluable to his chief. In regard to forms, the Army forms were mainly medical, for civilian methods of accountancy, in the main, continued. To supervise patients when in the grounds, three orderlies, being ex-members of the Metropolitan Police, were "badged," and became

* *The Story of the Horton (County of London) War Hospital, Epsom. Its inception and work, and some Reflections.* By Lieut.-Colonel J. R. Lord, C.B.E., M.B. With seven plates and plan. (London: W. Heinemann (Medical Books), Ltd. Price 12s. 6d. net; library edition, 15s. net.)

**A Mental Hospital Revolution**—(*continued*).

known as "hospital police," who carried on their persons a copy of their powers and duties.

Now these hints of the measures taken to make the needed change, which indicate the spirit in which it was attempted, suggest rather than recall what was being done. But, apart from the insight which they give into the problem, they deserve mention because of the opposition which the use of asylums as war hospitals aroused. This led to the sending of a deputation to the Secretary of State, to questions in Parliament, and so on. Consequently the medical superintendents were doubly put upon their mettle. They had a difficult problem, and had to undertake it in a somewhat critical atmosphere. So, while the word "Horton" was dropped out of the title, extra care was taken to give the soldiers no cause of complaint, and among the several causes for complacency which Colonel Lord found in his work were the admiration and gratitude expressed in the Epsom newspapers.

We have devoted this brief review to the preliminaries of the work, because in administrative record of change the "how" is more important than the "what." But the whole volume is excellent reading, and the work is not merely an institutional diary, but a very readable contribution to hospital history generally.

